# Human epidemiological data of Crimean-Congo haemorrhagic fever, 2014–2023: scoping review over one decade

**DOI:** 10.1016/j.onehlt.2026.101617

**Published:** 2026-10-13

**Authors:** Luca N. Zweers, Kay H.S. van Son, Alma Tostmann, Corien M. Swaan, Aura Timen, Chantal P. Rovers

**Affiliations:** a Department of Internal Medicine, Radboudumc Centre for Inflammation, Infection and Immunity, Radboudumc Community for Infectious Diseases, Radboud University Medical Centre, Postbus 9101, 6500 HB Nijmegen, the Netherlands; b Department of Medical Microbiology, Radboud Community for Infectious Diseases, Radboud University Medical Centre, Postbus 9101, 6500 HB Nijmegen, the Netherlands; c National Coordination Centre for Infectious Disease Control, National Institute for Public Health and the Environment, Postbus 1, 3720 BA, Bilthoven, the Netherlands; d Department of Primary and Community Care, Radboud University Medical Centre, Postbus 9101, 6500 HB, Nijmegen, the Netherlands

**Keywords:** Crimean-Congo haemorrhagic fever, Tick-borne diseases, Emerging infectious diseases, Epidemiology, One health, Zoonoses, Public health surveillance

## Abstract

Crimean-Congo haemorrhagic fever (CCHF), a severe emerging viral tick-borne disease, poses a global threat. The virus, endemic to Africa, the Middle East, Asia, and the Balkans, is being detected in new regions. This study aimed to summarise available human CCHF epidemiological data and determine whether scientific literature, surveillance system reports, and public health authorities' disease notifications offer uniform and sufficiently useful data to timely detect changes in epidemiological trends. We conducted a systematic PubMed search (2017–2023) using relevant CCHF MeSH and entry terms (e.g. CCHF and epidemiology). We reviewed ProMED reports and publicly available public health authorities' notifications on CCHF (2014–2023). We extracted case characteristics, reporting date, and transmission routes from these reports. The review includes 88 PubMed articles, 309 ProMED reports, and 44 public health authorities' notifications, reporting on 7073, 4169, and 3749 confirmed cases respectively, mostly from endemic regions. The number of ProMED reported cases did not increase significantly over one decade (*p* = .281). The case-fatality rate was significantly higher in the African and Southeast Asian Regions compared to the European and Eastern Mediterranean Regions. The risk appeared elevated in people working with animals and in healthcare workers. Tick exposure was the most frequently reported transmission route. None of the three data sources provided a complete overview of CCHF epidemiology. A more accurate CCHF monitoring system is needed to detect epidemiological changes timely. Addressing current gaps, such as inconsistencies in reporting frequency, requires coordinated, standardised data collection and sharing using standardised reporting frameworks to strengthen global preparedness and response.

## Highlights

• Crimean-Congo haemorrhagic fever is an emerging severe viral tick-borne disease.
• Literature, ProMED reports and public health notices report on its epidemiology.
• None of the three databases individually offered a complete overview of the disease.
• Standardised data collection and sharing important to monitor epidemiology trends.
• Data collection and sharing strengthens global outbreak preparedness and response.

## Graphical abstract

**Figure:**
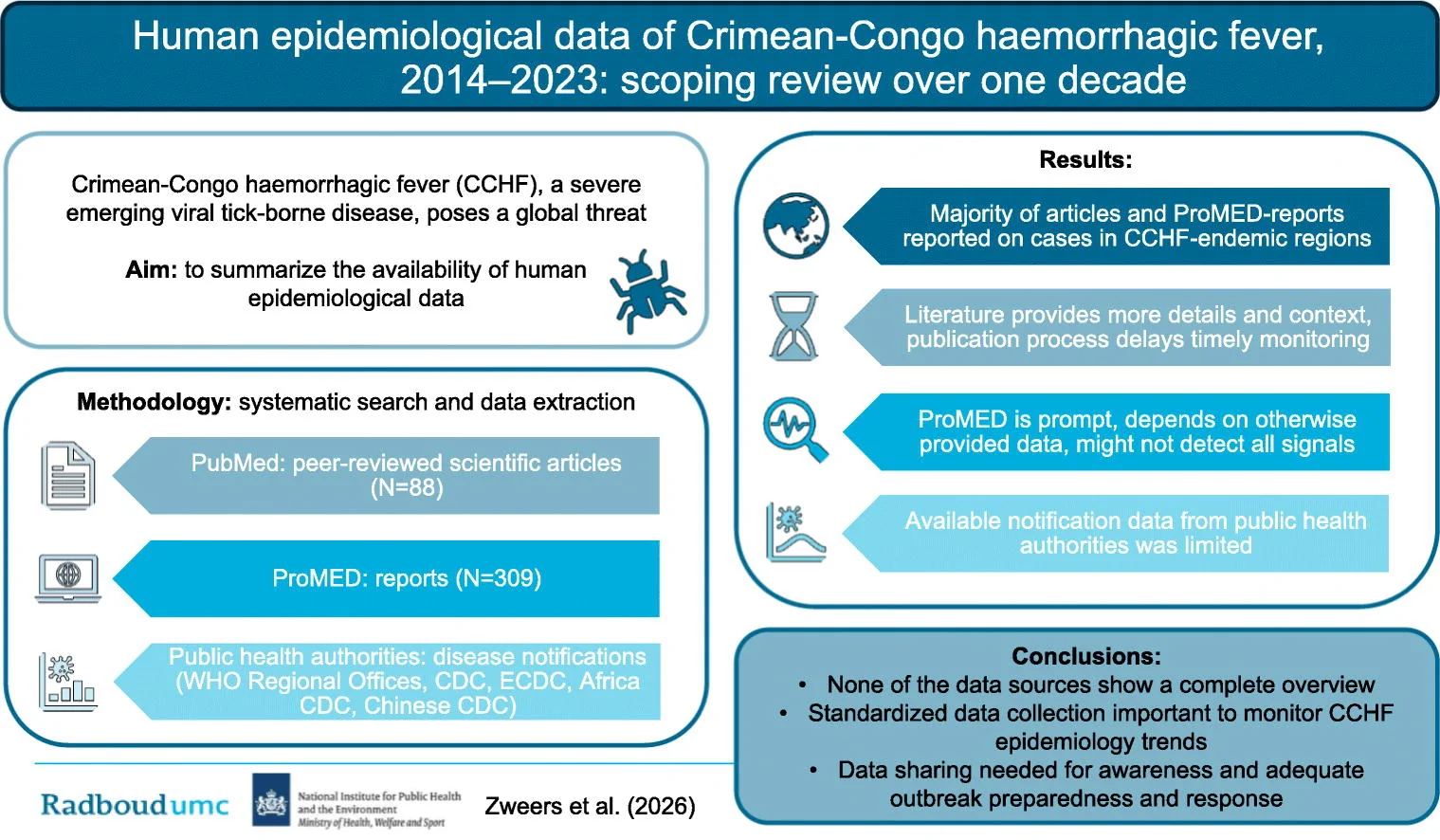

## 1 Introduction

Crimean-Congo haemorrhagic fever (CCHF) is a viral tick-borne disease caused by the CCHF virus (CCHFV) of the Nairoviridae family. The principal vectors are *Hyalomma* ticks*,* which feed on both livestock and humans. Transmission to humans occurs through direct contact with infected ticks, tick bites, or contact with infected bodily fluids or tissue of humans or animals. Numerous wild and domestic animals can host CCHFV such as cattle, sheep, and goats. Individuals in livestock-related occupations (e.g. farmers, agricultural workers, slaughterhouse workers, and veterinarians) as well as healthcare workers (HCW) are at increased risk. CCHF can cause mild to severe disease in humans. In severe cases, symptoms typically start within nine days from infection and include sudden onset of fever, myalgia, dizziness, headache, and haemorrhage. Severe illness often occurs five days after onset with kidney, liver, and/or respiratory failure. The case-fatality rate (CFR) varies between 10 and 40% during outbreaks and is approximately 30% among hospitalised patients [1].

CCHF is endemic in Africa, the Middle East, the Balkans, and in Asian countries south of the 50th parallel north [1], [2], and is now also considered endemic in Spain, where ten autochthonous cases have been notified between 2013 and 2021 [3]. In Europe, *Hyalomma marginatum* and *Hyalomma lusitanicum* are the principal tick species to transmit the virus to humans [4]. A warmer climate may facilitate expansion of the vector into Northern Europe [5]. CCHF is listed as one of the prioritised diseases by the World Health Organisation (WHO), which are diseases that pose a significant public health risk due to their epidemic potential and/or lack effective countermeasures [6]. Patients with a (suspected) CCHF infection are admitted to hospitals under very strict infection prevention and control measures, following similar guidelines as developed for Ebola and Marburg virus disease [1]. Dedicated isolation rooms and appropriate personal protective equipment (PPE) are necessary to ensure staff safety and prevent nosocomial transmission. No curative treatment is available, and management is primarily supportive. Ribavirin is often prescribed to decrease symptoms and improve outcome [1], [7], although its effectiveness in CCHF remains controversial [8], [9].

Monitoring the geographical spread of CCHFV and its vector is essential, as first detection in new areas requires preparedness and awareness in countries at-risk [10], [11]. Several reviews on CCHF exist, including research on regional epidemiology [12] and cross-border spread [13]. Nasirian conducted a systematic literature review including data from 1944 to 2016 [14], while Ince et al. summarised ProMED reports from 1998 to 2013 [15]. ProMED is the largest publicly-available surveillance system on global infectious diseases outbreaks, providing timely reporting of emerging and re-emerging pathogens. Regularly updated reviews of cases reported in literature and online surveillance tools are essential to identify changing epidemiological patterns.

We conducted a scoping review to summarise the available human epidemiological data from peer-reviewed scientific articles, ProMED reports, and available official notification data from public health authorities over one decade. Building on previous work, this review provides a novel contribution by combining and comparing epidemiological data from three distinct data sources. We determined whether these data sources offer uniform and sufficiently useful data to timely detect changes in epidemiological trends. The scoping review methodology provides us the opportunity to create a comprehensive overview of available epidemiological information, such as incidence, fatalities, and exposure routes, and to assess its potential regarding real-time monitoring of human CCHF data.

## 2 Methods

### 2.1 Literature

A systematic literature search was conducted in PubMed on October 9th, 2023. The search strategy combined relevant MeSH and entry terms related to CCHF and Epidemiology (Supplementary File S1). Peer-reviewed articles published between January 1st, 2017 until October 9th, 2023 were included to align our findings with previous research [14]. All retrieved articles were imported into Rayyan.ai, a web-based screening tool. Titles and abstracts were systematically screened for eligibility by the second author, uncertainties being resolved through readily consultation and discussion with the first author subsequently. Articles were included when reporting one or more of the following CCHF-related characteristics: human case reports or case series, seroprevalence studies, epidemiological studies, or descriptions of cases within broader research. Exclusion criteria were: reporting exclusively non-human CCHF cases, absence of epidemiological data, presenting only cases identified before 2014 (to avoid duplication with a previous systematic review [14]), systematic reviews (to prevent duplicate use of data), non-English articles, or no full-text available through Radboudumc's Medical Library. Full-text screening followed the same criteria. When the period of case collection was not reported, study authors were contacted to acquire this information. If no response was received and the period remained unknown, the article was excluded. Eligible articles were categorised according to WHO region definitions [16]. Full-texts of included articles were reviewed to extract data on CCHF cases.

### 2.2 ProMED reports

A systematic search in the ProMED surveillance system was conducted on October 9th, 2023 using the following search terms: “Crimean-Congo Haemorrhagic Fever”, “Crimean-Congo”, and “CCHF”. Full-text reports published between January 1st, 2014 and October 9th, 2023 were screened, this time period was selected to prevent overlap with previously published findings [15]. The second author reviewed all titles and abstracts, consulting the first author readily when clarification or discussion was needed. Reports were included if they contained information on confirmed human CCHF cases. Reports were excluded if they focused solely on disease characteristics or prevention, lacked individual case information, or described only suspected cases. All included reports were cross-checked to avoid duplication. When more than one report notified the same case(s) (e.g. follow-up report providing the cumulative number of cases for a specific year in a specific country), the total number of cases was extracted from the latest report.

### 2.3 Official notification data

We searched the websites of the WHO, WHO Regional Offices, the Centres for Disease Control and Prevention (CDC), European Centre for Disease Prevention and Control (ECDC), Chinese Centre for Disease Control and Prevention, and Africa Centre for Disease Control and Prevention for publicly available CCHF notification data from public health institutes. Using their websites' search function, we searched for “Crimean-Congo Haemorrhagic Fever”, “Crimean-Congo”, and “CCHF”. Notifications published between January 1st, 2014, and October 9th, 2023 were included.

### 2.4 Data extraction

Two researchers (first and second author) extracted the following variables from peer-reviewed articles, reports, and public health notification data: publication date, study period, country, number of confirmed cases, age, sex, hospitalisation, number of fatalities and CFR, occupation, and transmission route. Extracted data were summarised in Microsoft Excel and cross-checked by both researchers to ensure validity.

### 2.5 Data analysis

Descriptive analyses were performed using IBM SPSS Statistics (Version 29). For 2023, case numbers from ProMED and public health authorities' notifications were extrapolated. A Jonckheere–Terpstra test was applied to ProMED data to assess temporal trends. A chi-square test for independence was used to evaluate differences in CFR across the four WHO regions, followed by pairwise z-tests with Bonferroni correction to account for multiple comparisons.

## 3 Results

### 3.1 Included articles and reports

The PubMed search identified 292 unique peer-reviewed articles published between 1st of January 2017 and 9th of October 2023. Based on title and abstract screening, 159 articles were excluded according to predefined criteria. The remaining 133 identified articles underwent full-text reviewing, after which 45 articles were excluded. In total, 88 articles met the inclusion criteria and were analysed (Fig. 1). These articles were published in 2017 (*n* = 9), 2018 (*n* = 10), 2019 (*n* = 19), 2020 (*n* = 12), 2021 (*n* = 17), 2022 (*n* = 9), and 2023 (*n* = 12).

**Fig. 1:**
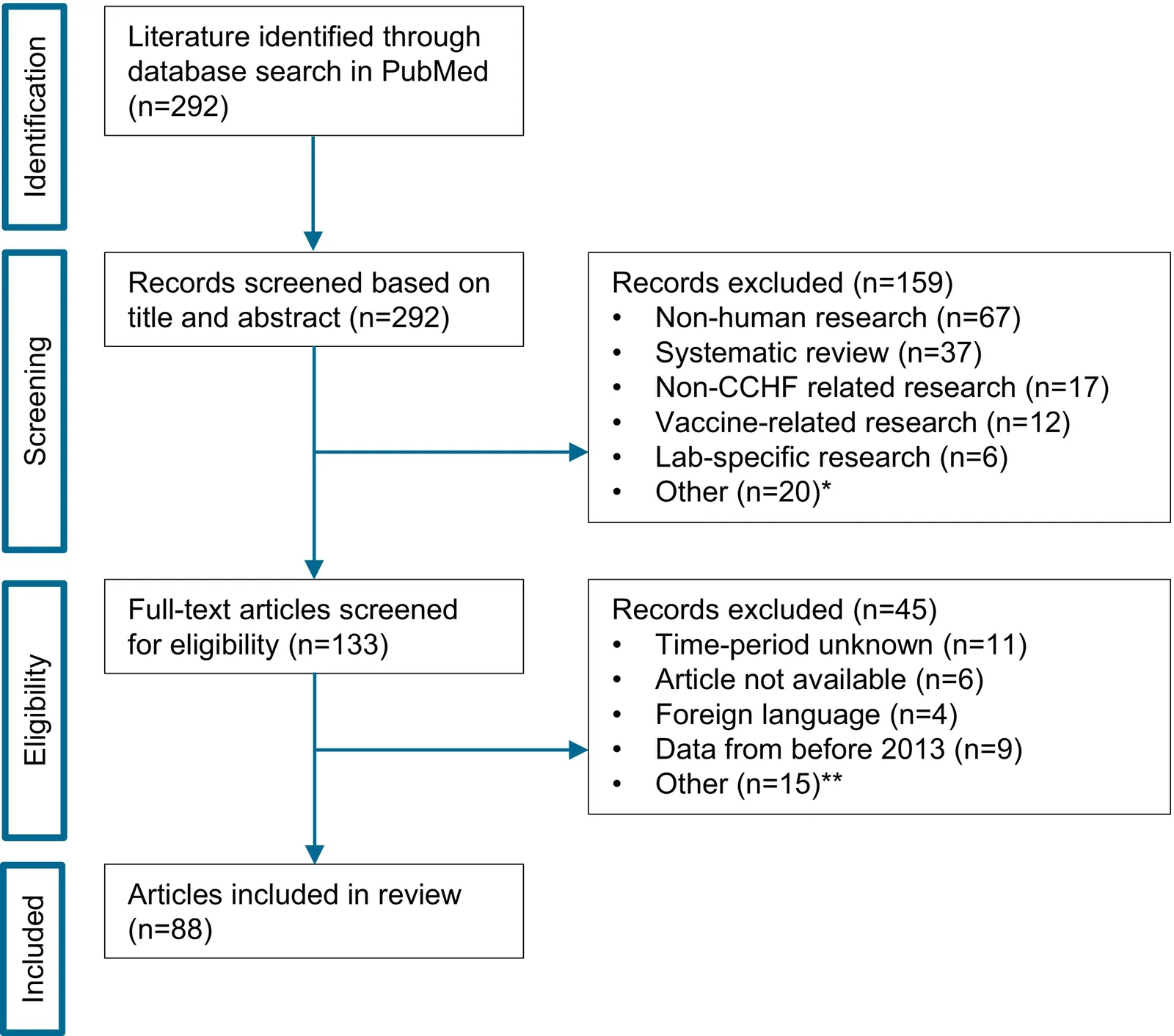
Flowchart of article identification and selection. *Other reasons for exclusion based on title and abstract are phylogenetic research, geographical research, research on costs, model research, background articles, no epidemiological data presented, and ProMED database research. ** Other reasons for exclusion by full-text are data overlap, background articles, no cases mentioned, and literature reviews.

The ProMED search identified 359 reports. During full-text screening, 21 reports were excluded because they solely described disease and prevention characteristics, and 29 reports were excluded because they solely described suspected cases. A total of 309 ProMED reports were included. In 86 reports, the same case(s) were described in a more recently published report. The number of cases was extracted from the most recent report, while additional details (e.g., gender, CFR, and transmission route) were extracted from earlier reports if not provided in the latest update.

Official notification data on CCHF were found on the website of ECDC, Africa CDC, WHO, and WHO Eastern Mediterranean Region (EMRO). ECDC has reported CCHF cases in the European Union (EU) member states since 2013 [17], while Africa CDC provides data dating back to 2003 [[18], [19], [20], [21]]. WHO reported one outbreak in Iraq in 2022 in a Disease Outbreak News [22]. WHO EMRO documented three CCHF-outbreaks in their region in 2023 [23]. No official notification data was found on the CDC, Chinese CDC, and remaining WHO Regional Offices websites.

### 3.2 Description of CCHF cases

7073 confirmed CCHF cases were reported in 88 articles published between January 1st, 2017, until October 9th 2023 (Table 1) [[24], [25], [26], [27], [28], [29], [30], [31], [32], [33], [34], [35], [36], [37], [38], [39], [40], [41], [42], [43], [44], [45], [46], [47], [48], [49], [50], [51], [52], [53], [54], [55], [56], [57], [58], [59], [60], [61], [62], [63], [64], [65], [66], [67], [68], [69], [70], [71], [72], [73], [74], [75], [76], [77], [78], [79], [80], [81], [82], [83], [84], [85], [86], [87], [88], [89], [90], [91], [92], [93], [94], [95], [96], [97], [98], [99], [100], [101], [102], [103], [104], [105], [106], [107], [108], [109], [110], [111]], including both individuals with a CCHF-positive PCR test and CCHF-seropositive individuals who either had a (recent) acute infection or a past infection. The age of the cases was reported in 2888 cases in 57 articles. The calculated mean age was 42.6 years (Supplementary File S2, Table S2.1). In 49 articles, 433 fatalities were reported over 2749 cases, resulting in an overall mean CFR of 15.8%. The mean CFR and CFR range per region are as follows: African region (20.9%, 0–100%), Eastern Mediterranean Region (20.7%, 0–66.7%), European Region (6.0%, 0–100%), and Southeast Asian Region (46.9%, 33.3–66.7%). The mean CFR and CFR range grouped per study type is depicted in Table S2.2 of the Supplementary Data. The hospitalisation rate ranged from 0 to 100%.

**Table 1:** Demographic data of CCHF-confirmed cases in the African Region, Eastern Mediterranean Region, Southeast Asian Region, and European Region reported in scientific articles published between 2017-Oct 2023 (*N* = 88).

WHO region	Country	Period	Cases (N)	Age (mean)	Sex (%male)	Hospitalisation (%)	Fatalities (N) (CFR)	Type of study	Reference
African Region	Guinea	2016–2019*	67	Unknown	46.3	Unknown	Unknown	Seroprevalence	[24]
	Kenya	17/06/2008–13/08/2015*	54	Unknown	Unknown	Unknown	Unknown	Seroprevalence	[25]
		01/2020–12/2021	29	Unknown	55.2	100	0 (0)	Seroprevalence	[26]
	Mali	04/2016–05/2017	2	1.5	100	100	Unknown	Seroprevalence	[27]
	Mauritania	17/06/2019–09/07/2019	2	52.5	100	100	0 (0)	Case report	[28]
		02/2022–05/2022	7	47	57.1	100	2 (28.6)	Contact investigation	[29]
	Mozambique	03/2015–03/2016	8	28	50	Unknown	Unknown	Seroprevalence	[30]
	Senegal	10/2020–11/2020	10	29	Unknown	Unknown	Unknown	Seroprevalence	[31]
		06/2021–03/2022	38	31	39.7	Unknown	Unknown	Seroprevalence	[32]
	South Africa	2012–2017	2	27	100	0	0 (0)	Seroprevalence	[33]
		05/2017–05/2018	21	Unknown	Unknown	Unknown	Unknown	Seroprevalence	[34]
		06/2017	1	62	100	100	1 (100)	Case report	[35]
	Tanzania	06/2018–11/2018	10	Unknown	40	60	Unknown	Seroprevalence	[36]
	Uganda	2013–2019	32	28	75	100	10 (31.3)	Case report review	[37]
		06/11/2015	1	33	100	100	0 (0)	Case report	[38]
		07/2018–01/2019	14	24	64	100	5 (35.7)	Outbreak investigation	[39]
		12/2018–03/2019	221	Unknown	82.8	Unknown	Unknown	Seroprevalence	[40]
		07/2019	1	42	100	100	1 (100)	Outbreak investigation	[41]
		03/2020–07/2020	49	Unknown	83.7	Unknown	Unknown	Seroprevalence	[42]
		04/2021	2	14.5	50	100	0 (0)	Outbreak investigation	[43]
	**Sub total (N (% of total))**		**571 (8.1)**						
Eastern- Mediterranean Region	Afghanistan	2016–2018	293	Unknown	68.5	Unknown	127 (43.3)	Retrospective epidemiology	[44]
		03/2017–12/2018	51	30	72.5	100	11 (21.6)	Retrospective epidemiology	[45]
		05/2017–10/2017	63	35.4	60.3	100	14 (22.2)	Outbreak investigation	[46]
		05/05/2018	1	30	0	100	0 (0)	Case report	[47]
	Iran	2000–2014	1027	Unknown	Unknown	Unknown	Unknown	Retrospective epidemiology	[48]
		2000–2016	161	15.5	70.8	Unknown	19 (11.8)	Seroprevalence	[49]
		2007–2017	160	32.9	80	Unknown	7 (4.4)	Case series	[50]
		2010–2017	190	31.9	88	Unknown	16 (8.4)	Ecology analysis	[51]
		2014	1	32	100	Unknown	Unknown	Seroprevalence	[52]
		06/2015–07/2015	3	Unknown	Unknown	100	1 (33.3)	Case series	[53]
		2017	73	Unknown	Unknown	Unknown	Unknown	Diagnostic test evaluation	[54]
	Iraq	2022–2023	545	Unknown	Unknown	Unknown	88 (16.2)	Epidemiological report	[55]
	Oman	2013–2017	80	Unknown	Unknown	Unknown	26 (32.5)	Retrospective epidemiology	[56]
	Pakistan	2015–2017	51	Unknown	45.1	Unknown	Unknown	Seroprevalence	[57]
		06/03/2016–24/06/2016	3	37.7	66	100	2 (66.7)	Case study analysis	[58]
		10/2016–05/2017	22	Unknown	72.7	Unknown	Unknown	Seroprevalence	[59]
		11/2016–03/2017	26	Unknown	84.6	Unknown	Unknown	Seroprevalence	[60]
		05/2017	6	Unknown	100	83.3	4 (66.7)	Outbreak investigation	[61]
		2019	28	37.3	92.8	100	11 (39.3)	Molecular epidemiology	[62]
		2022	10	Unknown	Unknown	Unknown	Unknown	Narrative	[63]
		2022	6	26	100	Unknown	3 (50)	Molecular epidemiology	[64]
	Sudan	09/2012–05/2016	7	22.3	100	Unknown	1 (14.3)	Retrospective cohort	[65]
		12/2012–01/2013	12	Unknown	Unknown	100	Unknown	Seroprevalence	[66]
		2015–2020	10	Unknown	Unknown	Unknown	Unknown	Retrospective epidemiology	[67]
	**Sub total (N (% of total))**		**2829 (40.0)**						
Southeast Asian Region	India	2013	12	43.4	66.7	100	4 (33.3)	Retrospective cohort	[68]
		2013–2019	14	Unknown	Unknown	Unknown	Unknown	Diagnostic test evaluation	[69]
		2014–2015	21	38	76.2	Unknown	Unknown	Retrospective cohort	[70]
		2015–2017	25	Unknown	72	Unknown	Unknown	Seroprevalence	[71]
		2019	3	43	66.7	100	2 (66.7)	Case report	[72]
		2019	34	42.3	61.8	100	17 (50)	Prospective cohort	[73]
	Myanmar	06/2016–08/2018	10	Unknown	60	Unknown	Unknown	Seroprevalence	[74]
	**Sub total (N (% of total))**		**119 (1.7)**						
European Region	Bulgaria	05/2015–10/2015	55	64	45.5	Unknown	Unknown	Seroprevalence	[75]
		05/2018	1	Unknown	100	100	0 (0)	Case report	[76]
	Georgia	10/2014–02/2016	4	Unknown	Unknown	Unknown	Unknown	Seroprevalence	[77]
	Hungary	2008–2017	10	34.6	80	0	Unknown	Seroprevalence	[78]
	Kazakhstan	04/2014–10/2015	106	35	60.4	Unknown	Unknown	Seroprevalence	[79]
		06/2017	17	54	70.8	Unknown	Unknown	Seroprevalence	[80]
	Kosovo	2013–2016	15	40	87	100	Unknown	Diagnostic test evaluation	[81]
	North – Macedonia	07/2023	2	Unknown	Unknown	100	1 (50)	Case report	[82]
	Russia	2007–2017	500	Unknown	Unknown	Unknown	Unknown	Retrospective epidemiology	[83]
		02/06/2016	2	13	50	100	1 (50)	Case report	[84]
		04/2018–06/2018	1	Unknown	Unknown	Unknown	Unknown	Seroprevalence	[85]
	Spain	2013	1	32	0	100	0 (0)	Case report	[86]
		01/08/2016	2	56	50	100	1 (50)	Case report	[87]
		2017–2018	3	40.7	100	0	Unknown	Seroprevalence	[88]
		10/2017–10/2018	4	64.3	75	100	Unknown	Seroprevalence	[89]
		31/07/2018	1	74	100	100	1 (100)	Case report	[90]
		2020–2021	5	56.4	80	Unknown	Unknown	Diagnostic test evaluation	[91]
	Türkiye	2002–2016**	1	20	0	100	0 (0)	Case series	[92]
		2008–2016	3	Unknown	Unknown	Unknown	Unknown	Retrospective epidemiology	[93]
		2010–2019	64	48.6	67	100	8 (12.5)	Retrospective epidemiology	[94]
		2010–2019	6	42.1	100	100	0 (0)	Retrospective epidemiology	[95]
		04/2010–08/2013	23	57	39.1	100	4 (17.4)	Retrospective epidemiology	[96]
		2011–2013	61	44.1	67.2	100	2 (3.3)	Case series	[97]
		2011–2017	206	53	50.5	100	7 (3.4)	Prospective epidemiology	[98]
		01/2011–12/2019	241	Unknown	54.5	100	8 (3.3)	Retrospective epidemiology	[99]
		2013–2021	931	47.6	56.6	Unknown	Unknown	Ecology analysis	[100]
		03/2013–07/2014	107	48	54.2	Unknown	9 (8.4)	Prospective cohort	[101]
		01/04/2013- 27/08/2023*	64	45.7	62.5	100	Unknown	Diagnostic test evaluation	[102]
		2014*	132	49.9	62.1	Unknown	13 (9.8)	Prospective cohort	[103]
		2014–2017	31	52.8	38.7	100	3 (9.7)	Epidemiology analysis	[104]
		27/05/2015–02/08/2015	65	53	62	100	2 (3.1)	Prospective cohort	[105]
		07/2015–08/2016	36	52.3	50	100	1 (2.8)	Prospective study	[106]
		06/2016	2	38	50	0	0 (0)	Seroprevalence	[107]
		2016–2017	775	Unknown	Unknown	Unknown	Unknown	Prediction study	[108]
		2019–2021	49	42.4	59.2	Unknown	Unknown	Diagnostic test evaluation	[109]
		04/2022–08/2022	27	38.9	63.0	100	0 (0)	Observational study	[110]
	United Kingdom	2014	1	70	100	Unknown	0 (0)	Narrative	[111]
	**Sub total (N (% of total))**		**3554 (50.2)**						
	**Total (N (%))**		**7073 (100)**						

* The time period was acquired through contact with the author. ** Only the case from Türkiye (2016) is included.

A total of 4169 confirmed CCHF cases were reported in 223 ProMED reports globally between January 1st, 2014, and October 9th, 2023 (Table 2; Supplementary File S2, Fig. S2.1). The number of reported cases did not increase significantly over the years (*p* = .281). With 524 confirmed fatalities, the overall CFR was 12.6%. The CFRs per region were as follows: African region 35.7%, Eastern Mediterranean Region 11.8%, European Region 11.1%, and Southeast Asian Region 47.4%. A significant variation in CFR was observed across the four regions (*p* < .001). The European and Eastern Mediterranean Regions showed a significantly lower CFR compared to the African and Southeast Asian Regions (Supplementary File S2, Table S2.3). The mean hospitalisation rate was 98%.

**Table 2:** ProMED reported confirmed CCHF cases per WHO region per country per year between 2014-October 2023, extrapolated for 2023 until December 31st, 2023*.

Period WHO region / Country	2014	2015	2016	2017	2018	2019	2020	2021	2022	2023	2023*	Total	Total*
**AFR total**	**1**	**1**	**–**	**18**	**18**	**10**	**18**	**2**	**10**	**6**	**7**	**84**	**85**
Mali	–	–	–	–	–	–	16	–	–	–	–	16	16
Mauritania	–	–	–	2	1	–	–	–	2	1	1	6	6
Namibia	1	–	–	3	2	3	–	–	–	1	1	10	10
Senegal	–	1	–	1	–	1	1	–	2	4	5	10	11
South Africa	–	–	–	2	–	3	–	–	3	–	–	8	8
Uganda	–	–	–	10	15	3	1	2	3	–	–	34	34
**EMR total**	**68**	**62**	**129**	**438**	**539**	**118**	**43**	**11**	**712**	**1523**	**1972**	**3643**	**4092**
Afghanistan	–	–	71	237	455	–	–	–	330	950	1230	2043	2323
Iran	–	9	–	120	56	54	38	–	78	60	78	415	433
Iraq	–	–	–	–	1	–	–	2	299	476	616	778	918
Oman	16	–	11	9	–	1	–	–	–	–	–	37	37
Pakistan	52	53	47	72	27	40	5	9	5	37	48	347	358
Sudan	–	–	–	–	–	22	–	–	–	–	–	22	22
UAE	–	–	–	–	–	1	–	–	–	–	–	1	1
**EUR total**	**41**	**101**	**2**	**–**	**2**	**38**	**20**	**31**	**110**	**59**	**77**	**404**	**422**
Bulgaria	–	–	–	–	–	–	1	–	–	–	–	1	1
Georgia	13	–	–	–	–	–	–	1	45	12	16	71	75
Greece	–	–	–	–	1	–	–	–	–	–	–	1	1
Kazakhstan	4	2	–	–	–	–	1	–	34	3	4	44	45
North Macedonia	–	–	–	–	–	–	–	–	–	3	4	3	4
Russia	22	97	–	–	–	38	1	14	1	–	–	173	173
Spain	–	–	2	–	1	–	2	2	2	–	–	9	9
Türkiye	1	2	–	–	–	–	15	14	27	41	53	100	112
UK	1	–	–	–	–	–	–	–	1	–	–	2	2
**SEAR total**	**3**	**11**	**5**	**2**	**1**	**9**	**4**	**–**	**–**	**3**	**4**	**38**	**39**
India	3	11	5	2	1	9	4	–	–	3	4	38	39
**Total**	**113**	**175**	**136**	**458**	**560**	**175**	**85**	**44**	**832**	**1591**	**2059**	**4169**	**4638**

AFR: African Region, EMR: Eastern Mediterranean Region, EUR: European Region, SEAR: Southeast Asian Region, UAE: United Arab Emirates, UK: United Kingdom.

*Publications from the year 2023 were only included until the 9th of October. For the columns with asterisk (2023* and total*) the total number of cases is extrapolated to make a proportional comparison.

ECDC reported 46 confirmed cases (CFR = 19.6%) in the EU and Africa CDC reported on 14 confirmed cases (CFR = 35.7%) in African countries between 2014 and October 9th, 2023 (Table 3). No information on age was provided. In the outbreak in Iraq in 2022, the Republic of Iraq notified 97 confirmed cases (CFR = 13.4%). WHO EMRO documented three outbreaks in 2023: Afghanistan (*N* = 1264, CFR = 9.0%), Iraq (*N* = 2219, CFR = 6.5%) and Pakistan (*N* = 109, CFR = 27.5%). No other details, such as transmission route, were publicly available.

**Table 3:** Notified confirmed cases of CCHF per country per year who were infected in the EU/EEA, African Region, and Eastern Mediterranean Region as published by ECDC [17], African CDC [18], [19], [20], and WHO EMRO [[22], [23]] respectively between 2014-October 2023, extrapolated for 2023 until December 31st, 2023*.

Period WHO region / Country	2014	2015	2016	2017	2018	2019	2020	2021	2022	2023	2023*	Total	Total*
**AFR total**	**–**	**1**	**–**	**–**	**4**	**–**	**–**	**–**	**8**	**1**	**1**	**14**	**14**
Mauritania	–	–	–	–	–	–	–	–	3	–	–	3	3
Senegal	–	1	–	–	–	–	–	–	1	1	1	3	3
South Africa	–	–	–	–	–	–	–	–	2	–	–	2	2
Uganda	–	–	–	–	4	–	–	–	2	–	–	6	6
**EMR total**	**–**	**–**	**–**	**–**	**–**	**–**	**–**	**–**	**97**	**3592**	**4649**	**3689**	**4746**
Afghanistan	–	–	–	–	–	–	–	–	–	1264	1636	1264	1636
Iraq	–	–	–	–	–	–	–	–	97	2219	2872	2316	2969
Pakistan	–	–	–	–	–	–	–	–	–	109	141	109	141
**EUR total**	**9**	**4**	**6**	**2**	**9**	**2**	**4**	**2**	**4**	**4**	**5**	**46**	**47**
Bulgaria	8	4	4	2	6	2	1		2	3	4	32	33
Greece	–	–	–	–	1	–	–	–	–	–		1	1
Spain	–	–	2	–	2	–	3	2	2	1	1	12	12
UK**	1	–	–	–	–	–	N/A	N/A	N/A	N/A		1	1
**Total**	**9**	**5**	**6**	**2**	**13**	**2**	**4**	**2**	**109**	**3597**	**4656**	**3749**	**4808**

AFR: African Region, EMR: Eastern Mediterranean Region, EUR: European Region, UK: United Kingdom, N/A: not applicable.

*Publications from the year 2023 were only included until the 9th of October. For the columns with asterisk (2023* and total*) the total number of cases is extrapolated to make a proportional comparison.

*Since 2020, the UK is no longer a member of the EU.

### 3.3 Geography

Of the 88 included articles, 79 (89.8%) focused on CCHF-endemic regions. Most articles focused on the European Region (*n* = 37), accounting for 3554 out of 7073 cases (50.2%). No studies addressed the Region of the Americas, or the Western Pacific Region. Türkiye was the most frequently studied country (*n* = 19), with a total of 2824 confirmed cases reported in literature.

Most CCHF cases reported by ProMED (95.5%) occurred in CCHF-endemic regions. 3643 of 4169 cases (87.4%) originated from the Eastern Mediterranean Region (Table 2). Most cases were reported in Afghanistan: 2043 cases over 5 years.

Table 3 depicts the countries on which the public health authorities reported cases. The case from the UK was imported, infected via a tick bite during a stay in Bulgaria.

### 3.4 Routes of transmission

Twenty-three articles reported transmission route in 1288 people (Table 4). The most frequently reported transmission route was tick exposure (*n* = 703, 54.6%) and contact with animals and/or livestock (*n* = 421, 32.7%). Vertical transmission was reported in two articles describing two pregnant women with CCHF. Both women survived, but the disease was fatal for one foetus and one neonate [[84], [92]].

**Table 4:** Reported routes of transmission in cases where the transmission route is known⁎ for cases described in scientific literature (*N* = 1288) and by ECDC (*N* = 43) [17] between 2014-October 2023.

	In scientific literature reported cases (N, (%))	By ECDC reported cases (N, (%))
Tick exposure	703 (54.6)	42 (97.7)
Tick bites	564/703 (80.2)	42/42 (100)
Gripping/crushing tick	8/703 (1.1)	–
Not specified	131/703 (18.6)	–
Contact with animals and/or livestock	421 (32.7)	–
Contact with animal bodily fluids and/or tissue	159 (12.3)	–
Contact with human bodily fluids and/or tissue	5 (0.4)	1 (2.3)
Total	1288 (100)	43 (100)

⁎ 99 individuals reported in scientific literature and 4 individuals reported by ECDC were exposed to ticks as well as animals and/or livestock, only the tick exposure is depicted in the table.

In 40 ProMED reports, the route of transmission was reported, of which 35 mentioned a history of tick bites or contact with body fluids of animals.

In 42 out of 46 cases (91.3%) reported by ECDC, the persons were infected through infected tick bites. One person was infected through nosocomial transmission (2.2%). In three people the mode of transmission was unknown (6.5%) (Table 4). The Africa CDC and WHO did not report transmission route.

### 3.5 Occupation

Occupation was reported in 26 articles. Livestock-related professions were most prevalent: farmers, animal breeders, abattoir workers, and shepherds were mentioned in 21 (80.8%) articles [[26], [28], [29], [33], [34], [35], [37], [38], [41], [42], [45], [46], [47], [51], [52], [60], [64], [72], [80], [98], [109]]. Five articles described an infection among HCW (*n* = 10) [[70], [82], [87], [93], [107]].

Occupation of cases was reported in 58 ProMED reports (17.2%), again most commonly involving livestock-related work. Nosocomial infections were described in ten HCW.

In the publicly available notification ECDC data, the occupation was specified for two cases: one forester and one HCW. The Africa CDC reported that one case was a butcher.

### 3.6 Healthcare setting

In literature, ten infections of CCHF among HCW were reported across Spain, Türkiye, North Macedonia, and India. Transmission occurred through improper removal of PPE (*n* = 1) [82], lack of PPE because CCHF was initially not suspected (*n* = 1) [87], accidents with needles or sharps (*n* = 3) [93], and percutaneous contact (*n* = 2) [107]. For three cases, the transmission route was not noted [70]. The disease was fatal in one HCW.

In the ProMED reports, ten infections among HCW were reported in Uganda, India, Pakistan, Spain, and North Macedonia between 2014 and 2023. The cause of infection was linked to inadequate or no use of PPE (*n* = 2) and direct contact with blood of the patient (*n* = 1) or not reported (*n* = 7).

In the ECDC data, one infection in a Spanish HCW, who made full recovery, was reported.

### 3.7 Seroprevalence

Twenty-nine seroprevalence studies in varying populations were included [[24], [25], [26], [27], [30], [31], [32], [33], [34], [36], [40], [42], [49], [52], [57], [59], [60], [66], [71], [74], [75], [77], [78], [79], [80], [85], [88], [89], [107]]. Among 7073 identified cases, 1021 people were retrospectively identified through CCHFV antibodies. Seroprevalence in at-risk groups (e.g. farmers and abattoir workers) ranged from 0.4 to 27.6%, while studies in not at-risk groups reported0.2–17.7%. Neither ProMED reports nor public health provided seroprevalence data.

## 4 Discussion

Combining data from scientific literature, ProMED reports, and public health notifications, we found that none of the three data sources provides a complete overview of CCHF occurrence (Fig. 2). Literature provides generally more details and context, but the delay inherent to the publication process make it unsuitable for timely monitoring epidemiology of emerging infectious diseases. Regional differences in awareness, laboratory capacity, and research focus, such as in Türkiye, may lead to underrepresentation in other areas. Online notification platforms, like ProMED, are emerging and re-emerging disease oriented. While the publication is comprehensive and prompt, it depends on otherwise provided data and might not detect all signals. ProMED, operated by the non-governmental, non-profit International Society for Infectious Diseases, has faced sustainable funding issues. Since July 2023, only posts from the previous 30 days are accessible, limiting retrospective review of reports published after October 2023. This short-term availability of data may hinder the ability to obtain a comprehensive overview in the future if such information is not documented elsewhere.

**Fig. 2:**
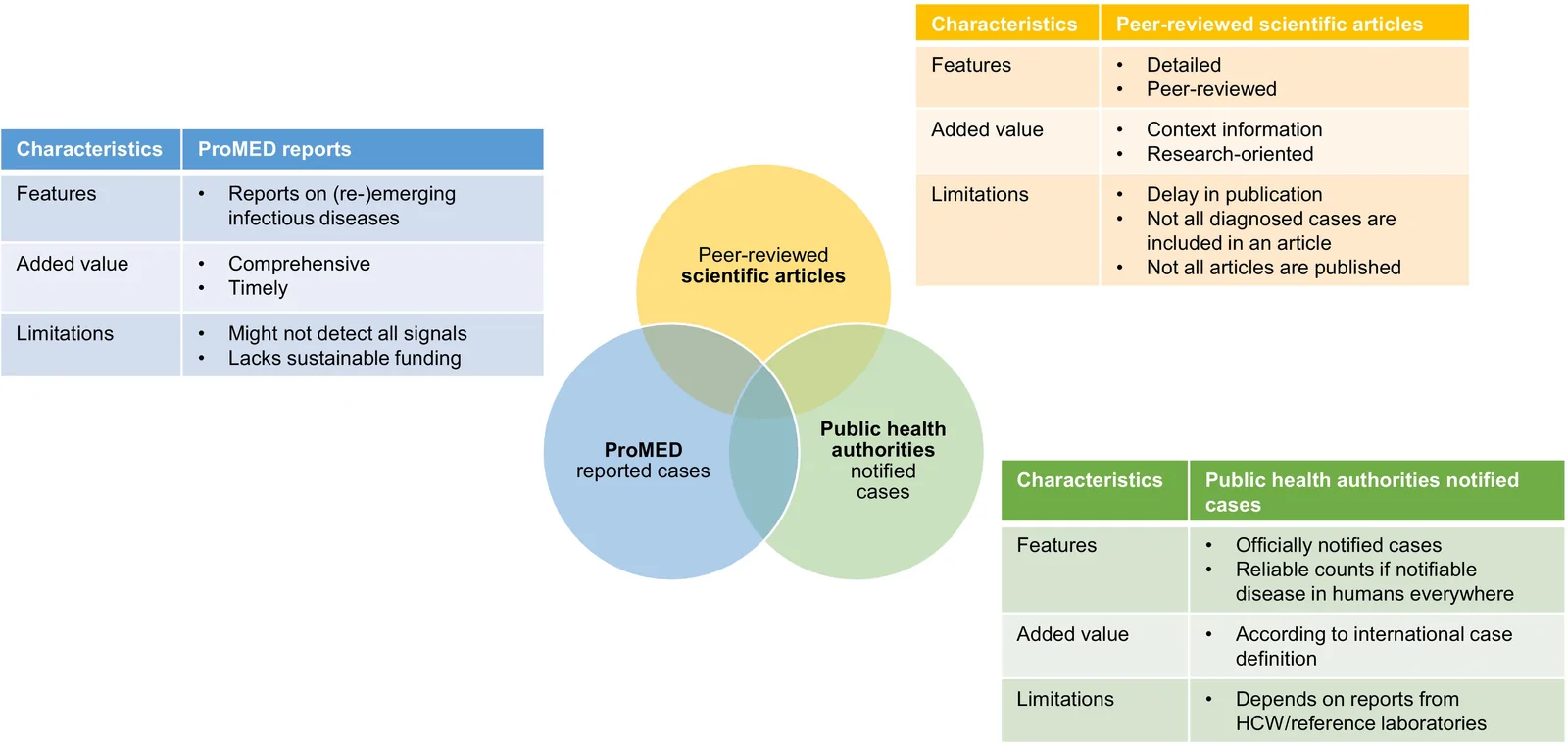
Overview of the features, added value and limitations of the three different data sources for CCHF case information used in this review: peer-reviewed articles, ProMED reports, and public health authorities' notifications. HCW: healthcare worker.

In many countries, public health authorities are responsible for providing an overview of notified cases and initiating appropriate actions. CCHF is a notifiable disease in the United States and the EU [[2], [112]], but a clear, accessible overview whether this applies to remaining countries as well is lacking. Official notifications generally follow international case definitions but rely on HCW and laboratories to notify cases to their Ministry of Health or public health authority. Therefore, completeness of notifications probably varies between countries. In this review, the three data sources complement each other, enhancing our understanding of CCHF epidemiology over the past decade, but providing and updating this combined overview is labour-intensive. The capture-recapture methodology using these sources may help estimate the true number of CCHF cases in this period, but must be applied cautiously, as individual-level information was not always available to determine overlapping cases across sources. Our findings suggest that overlap is inconsistent, reflected by the variation in case counts across the three data sources. Addressing the gap in literature-derived data for 2014–2016, Nasirian reports an estimated 200–250 cases annually during these years [14], suggesting that the total number of cases identified in literature would be even higher if these years were fully represented.

The number of CCHF cases reported by ProMED did not increase significantly over the past decade. A striking drop occurred in 2020 and 2021, which may have hidden a significant temporal increase. This decrease was most likely related to the COVID-19 pandemic: reduced human exposure, reduced healthcare seeking behaviour, reduced testing, and potential misdiagnosis [113]. In 2023, reported CCHF cases rose drastically compared with previous years, largely driven by the substantial outbreak in Afghanistan, also documented by the WHO EMRO.

The literature shows a remarkable high number of CCHF cases in the European region, attributable by numerous publications from Türkiye. A previous review also identified a high number of cases in the European region when classifying Türkiye within the WHO-defined European region [14]. In contrast, our ProMED data identified most cases in the Eastern Mediterranean region.

The ECDC data aligned with ProMED data for two of four EU countries. ProMED reported fewer cases in Spain and Bulgaria compared to the ECDC. ProMED reported more cases in the African region than the notifications findable on the website of Africa CDC. The opposite appeared in the Eastern Mediterranean region, where WHO EMRO reported higher numbers of cases in Afghanistan, Iraq, and Pakistan in 2023 than ProMED. ProMED's primary aim is early detection of infectious disease threats to warn health professionals timely, not replacement of formal surveillance systems [114]. Although WHO EMRO does provide outbreak overviews for 2023 [23], it offers limited information on previous years and other countries in their region. For example, cases were identified in Iran in both PubMed and ProMED, yet WHO does not publicly report these, although CCHF is a notifiable disease in Iran [115].

The CFR observed in our ProMED-analysis was comparable to a previous similar study [15] and within reported outbreak ranges [1]. The CFR was calculated only from cases with confirmed fatal outcomes. However, the true CFR may be underestimated, as ProMED reports often lack follow-up information, and clinical outcomes may be unknown at the time of posting. The ProMED acquired CFR in the European Region was significantly lower compared to the African and Southeast Asian Regions. In literature, the CFR in the European region appears lower than in other regions. This may reflect better access to and level of healthcare, and a higher level of awareness and response in this region, suggesting a true lower CFR in the European region. Alternatively, a higher likelihood of notifying less severe cases in the European region could increase the denominator and lower the CFR [116]. Furthermore, we found a high hospitalisation rate in both literature and ProMED reports. This likely reflects more severe cases being hospitalised and thus diagnosed and notified, whereas mild and/or asymptomatic cases might not seek care or might not be recognized as CCHF, resulting in underdiagnosis.

Tick exposure and contact with livestock and/or animals are the most frequently reported source of infection, emphasising the zoonotic nature of CCHF and the need for a One Health approach to manage CCHF adequately. These findings align with earlier findings [14] [15]. Seroprevalence studies suggest higher antibody positivity in at-risk populations, although estimates vary widely. This heterogeneity may reflect differing risk factors, such as geographical variation between endemic and non-endemic areas, and urban and rural areas [117], or to the variation across test systems, influenced by differences in technique and used antigens [118]. The seroprevalence between different regions could not be compared as the populations differed substantially. Awareness among HCW and people working with livestock remains crucial for timely recognition, and use of appropriate protective measures. Evidence from Ebola virus disease management highlights the importance of PPE and training for high-consequence infectious diseases, including CCHF [119]. Strengthening awareness and preparedness is essential for effective response, particularly in at-risk groups, endemic regions, and countries where CCHF may emerge.

Placing our findings in a wider public health context, the changes in vector dynamics and spread must be acknowledged. Although the likelihood of introduction and spread of CCHFV in CCHF-free countries in Europe was considered low in 2021 [120], the vectors were already widespread in Southwest Europe [121]. Climate change expands the geographical area in which the *Hyalomma* tick can establish [122]. While we did not observe a significant increase in cases over one decade, scientific literature, ECDC, and ProMED report numerous autochthonous human infections in Spain in the last decade, and recently Portugal reported its first autochthonous case since 1985 [123]. In France, the *Hyalomma* tick has been detected and CCHF-specific antibodies have been identified in domestic and wild animals [124]. While, up to 2023, no CCHF cases have been reported from France across any of the three data sources, locally acquired infections can be expected in France in the coming years.

This study's strength is the use of three different data sources, thereby providing a comprehensive perspective on human CCHF epidemiology. The three data sources are partly overlapping but provide a complementary perspective in terms of case numbers and characteristics, and transmission routes. Inclusion of all global regions provides a comprehensive overview. Furthermore, the study builds upon prior research, maintaining continuity in CCHF research.

A limitation of our study is the wide variety of study periods across the included scientific articles, which reduced the ability to make an accurate epidemiological overview. Some articles from the same country covered overlapping periods, but it was not possible to identify whether they described the same cases. Consequently, some cases may have been counted twice. Conversely, there has been underreporting in scientific articles, as not all CCHF cases lead to or are included in a scientific publication. Therefore, the results may not represent the true number of unique cases reported in literature. Additionally, seroprevalence studies may include individuals with mild or asymptomatic past infections. It is unknown whether these cases are not notified because they were asymptomatic or mild, or more severely ill but not recognized as CCHF in healthcare facilities. The differences between populations introduce potential bias in interpretating data from literature. For the literature published in 2014–2016, we relied on the findings from a previous review [14] and have not repeated the literature search for this time period. This means that relevant studies published before 2017 but indexed later may therefore be missing, contributing to underestimation. Additional limitations include the use of a single literature database, restriction to English-language publications, and screening by one reviewer, which may have led to missed studies. Additionally, our analysis with published data does not reflect true case numbers, with potential underrepresentation in regions with limited infrastructure and/or awareness. Finally, our inclusion period ended on October 9th, 2023, missing late 2023-cases, although proportional comparison was used to minimise this effect.

In conclusion, our findings emphasise the need for collaborative, standardised global data collection on clinical CCHF cases to monitor trends and changes in epidemiology of this severe disease. With the vector *Hyalomma* emerging in new areas, posing a global health threat, a more accurate, complete, and timely overview of cases is important to detect changes in geographic distribution and temporal incidence. The variation in case counts across and the not always available individual-level information in the three sources highlight limitations in current surveillance and reporting systems. ProMED has become a more challenging source to use due to policy changes, underscoring the need for alternative surveillance approaches. A common reporting framework would improve the clarity and comparability of available data. If not yet, CCHF should therefore be a notifiable disease globally in humans and additionally requires reporting of cases of antibodies or viremia detected in animals. However, it starts with early recognition of the disease and access to diagnostics. Therefore, increased knowledge on and training of CCHF among HCW, and appropriate facilities are crucial for timely recognition and diagnosis, and consequently safe patient care. When notifiable, health professionals must report CCHF cases to their Ministry of Health or national health authority, who in turn have the responsibility to share these data to enhance awareness, support accurate global assessment of CCHF epidemiology, and improve outbreak preparedness and response.

## CRediT authorship contribution statement

**Luca N. Zweers:** Visualization, Methodology, Investigation, Conceptualization, Writing – review & editing, Writing – original draft. **Kay H.S. van Son:** Visualization, Methodology, Investigation, Conceptualization, Writing – review & editing, Writing – original draft. **Alma Tostmann:** Visualization, Supervision, Methodology, Conceptualization, Writing – review & editing. **Corien M. Swaan:** Visualization, Supervision, Methodology, Conceptualization, Writing – review & editing. **Aura Timen:** Visualization, Supervision, Methodology, Conceptualization, Writing – review & editing. **Chantal P. Rovers:** Visualization, Supervision, Methodology, Conceptualization, Writing – review & editing.

## Declaration of competing interest

The authors declare that they have no known competing financial interests or personal relationships that could have appeared to influence the work reported in this paper.

## Data Availability

Data will be made available on request.

## References

[1] World Health Organisation. Crimean-Congo Haemorrhagic Fever 2022 [internet]. WHO [cited 2024 Jan 22]. Available from: https://www.who.int/news-room/fact-sheets/detail/crimean-congo-haemorrhagic-fever.

[2] Centres for Disease Control and Prevention (2024). https://www.cdc.gov/crimean-congo-hemorrhagic/about/index.html.

[3] Juanes H.M.L., Carbonell C., Sendra B.F., López-Bernus A., Bahamonde A., Orfao A. (2023). Crimean-Congo hemorrhagic fever, Spain, 2013-2021. Emerg. Infect. Dis..

[4] European Centre for Disease Prevention and Control (2023). https://www.ecdc.europa.eu/sites/default/files/documents/crimean-congo-haemorrhagic-fever-spatial-distribution-december-2023.pdf.

[5] Estrada-Peña A., D’Amico G., Fernández-Ruiz N. (2021). Modelling the potential spread of *Hyalomma marginatum* ticks in Europe by migratory birds. Int. J. Parasitol..

[6] World Health Organisation (2024). Pathogens prioritization: a scientific framework for epidemic and pandemic research preparedness. R&D Blueprint. https://cdn.who.int/media/docs/default-source/consultation-rdb/prioritization-pathogens-v6final.pdf?sfvrsn=c98effa7_9&download=true.

[7] Fletcher T.E., Gulzhan A., Ahmeti S., Al-Abri S.S., Asik Z., Atilla A. (2017). Infection prevention and control practice for Crimean-Congo hemorrhagic fever—a multi-center cross-sectional survey in Eurasia. PloS One.

[8] Johnson S., Henschke N., Maayan N., Mills I., Buckley B.S., Kakourou A. (2018). Ribavirin for treating Crimean Congo haemorrhagic fever. Cochrane Database Syst. Rev..

[9] Espy N., Pérez-Sautu U., De Arellano E.R., Negredo A., Wiley M.R., Bavari S. (2018). Ribavirin had demonstrable effects on the Crimean-Congo Hemorrhagic Fever Virus (CCHFV) population and load in a patient with CCHF infection. J. Infect. Dis..

[10] Leblebicioglu H., Ozaras R., Irmak H., Sencan I. (2016). Crimean-Congo hemorrhagic fever in Turkey: current status and future challenges. Antiviral Res..

[11] Sharififard M., Alavi S.M., Salmanzadeh S., Safdari F., Kamali A. (2016). Epidemiological survey of Crimean-Congo hemorrhagic fever (CCHF), a fatal infectious disease in Khuzestan Province, southwest Iran, during 1999–2015. Jundishapur J. Microbiol..

[12] Temur A.I., Kuhn J.H., Pecor D.B., Apanaskevich D.A., Keshtkar-Jahromi M. (2021). Epidemiology of Crimean-Congo hemorrhagic fever (CCHF) in Africa-underestimated for decades. Am J Trop Med Hy..

[13] Dreshaj S., Ahmeti S., Ramadani N., Dreshaj G., Humolli I., Dedushaj I. (2016). Current situation of Crimean-Congo hemorrhagic fever in Southeastern Europe and neighboring countries: a public health risk for the European Union?. Travel Med. Infect. Dis..

[14] Nasirian H. (2020). New aspects about Crimean-Congo hemorrhagic fever (CCHF) cases and associated fatality trends: a global systematic review and meta-analysis. Comp. Immunol. Microbiol. Infect. Dis..

[15] Ince Y., Yasa C., Metin M., Sonmez M., Meram E., Benkli B. (2014). Crimean-Congo hemorrhagic fever infections reported by ProMED. Int. J. Infect. Dis..

[16] World Health Organisation (2023). https://apps.who.int/violence-info/Countries%20and%20areas%20by%20WHO%20region%20-%2012bfe12.pdf.

[17] European Centre for Disease Prevention and Control (2023). https://www.ecdc.europa.eu/en/infectious-disease-topics/crimean-congo-haemorrhagic-fever/surveillance-and-updates/local-transmission-eueea-previous-years.

[18] Africa Centres for Disease Control and Prevention (2019). https://africacdc.org/disease/crimean-congo-haemorrhagic-fever/.

[19] Africa Centres for Disease Control and Prevention (2023). https://africacdc.org/download/africa-cdc-weekly-event-based-surveillance-report-may-2023/.

[20] Africa Centres for Disease Control and Prevention (2025). https://africacdc.org/speech/talking-points-weekly-press-briefing-on-covid-19-in-africa-thursday-th-october-2022/.

[21] Africa Centres for Disease Control and Prevention (2025). https://africacdc.org/speech/talking-points-weekly-press-briefing-on-covid-19-in-africa-thursday-13th-october-2022/#_heading=h.9vo123s8zldo.

[22] World Health Organisation (2023). https://www.who.int/emergencies/disease-outbreak-news/item/2022-DON386.

[23] WHO Regional Office for the Eastern Mediterranean (2023). https://www.emro.who.int/pandemic-epidemic-diseases/outbreaks/index.html.

[24] Naidenova E.V., Zakharov K.S., Kartashov M.Y., Agafonov D.A., Senichkina A.M., Magassouba N.F.F. (2020). Prevalence of Crimean-Congo hemorrhagic fever virus in rural areas of Guinea. Ticks Tick Borne Dis..

[25] Nyataya J., Maraka M., Lemtudo A., Masakhwe C., Mutai B., Njaanake K. (2020). Serological evidence of yersiniosis, tick-borne encephalitis, West Nile, Hepatitis E, Crimean-Congo hemorrhagic fever, lyme borreliosis, and brucellosis in febrile patients presenting at diverse hospitals in Kenya. Vector Borne Zoonotic Dis..

[26] Omoga D.C.A., Tchouassi D.P., Venter M., Ogola E.O., Osalla J., Kopp A. (2023). Transmission dynamics of Crimean–Congo haemorrhagic fever virus (CCHFV): evidence of circulation in humans, livestock, and rodents in diverse ecologies in Kenya. Viruses.

[27] Baumann J., Knüpfer M., Ouedraogo J., Traoré B.Y., Heitzer A., Kané B. (2019). Lassa and Crimean-Congo hemorrhagic fever viruses, Mali. Emerg. Infect. Dis..

[28] Boushab B.M., Kelly M., Kébé H., Bollahi M.A., Basco L.K. (2020). Crimean-Congo hemorrhagic fever, Mauritania. Emerg. Infect. Dis..

[29] Boushab B.M., Yanogo P.K., Barry D., Benane H.A., El Bara A., Abdellah M. (2022). Investigation around cases of Crimean-Congo hemorrhagic fever - Mauritania, 2022. Open Forum Infect. Dis..

[30] Muianga A.F., Watson R., Varghese A., Chongo I.S., Ali S., Monteiro V. (2017). First serological evidence of Crimean-Congo haemorrhagic fever in febrile patients in Mozambique. Int. J. Infect. Dis..

[31] Sankhe S., Talla C., Thiam M.S., Faye M., Barry M.A., Diarra M. (2023). Seroprevalence of Crimean-Congo hemorrhagic fever virus and rift valley fever virus in human population in Senegal from October to November 2020. IJID Reg..

[32] Mhamadi M., Badji A., Dieng I., Gaye A., Ndiaye E.H., Ndiaye M. (2022). Crimean–Congo hemorrhagic fever virus survey in humans, ticks, and livestock in Agnam (Northeastern Senegal) from February 2021 to March 2022. Trop. Med. Infect. Dis..

[33] Vawda S., Goedhals D., Bester P.A., Burt F. (2018). Seroepidemiologic survey of Crimean-Congo hemorrhagic fever virus in selected risk groups, South Africa. Emerg. Infect. Dis..

[34] Msimang V., Weyer J., Roux C.L., Kemp A., Burt F.J., Tempia S. (2021). Risk factors associated with exposure to Crimean-Congo haemorrhagic fever virus in animal workers and cattle, and molecular detection in ticks, South Africa. PLoS Negl. Trop. Dis..

[35] Krüger H.J., De Wet R., Blumberg L.H., Weyer J., Lemke F.G. (2019). Atypical presentation of Crimean-Congo haemorrhagic fever: lessons learned. S. Afr. Med. J..

[36] Rugarabamu S., Mwanyika G.O., Rumisha S.F., Sindato C., Lim H.Y., Misinzo G. (2021). Seroprevalence and associated risk factors of selected zoonotic viral hemorrhagic fevers in Tanzania. Int. J. Infect. Dis..

[37] Balinandi S., Whitmer S., Mulei S., Nyakarahuka L., Tumusiime A., Kyondo J. (2021). Clinical and molecular epidemiology of Crimean-Congo hemorrhagic fever in humans in Uganda, 2013–2019. Am. J. Trop. Med. Hyg..

[38] Balinandi S., Patel K., Ojwang J., Kyondo J., Mulei S., Tumusiime A. (2018). Investigation of an isolated case of human Crimean–Congo hemorrhagic fever in Central Uganda, 2015. Int. J. Infect. Dis..

[39] Mirembe B.B., Musewa A., Kadobera D., Kisaakye E., Birungi D., Eurien D. (2021). Sporadic outbreaks of Crimean-Congo haemorrhagic fever in Uganda, July 2018–January 2019. PLoS Negl. Trop. Dis..

[40] Atim S.A., Ashraf S., Belij-Rammerstorfer S., Ademun A.R., Vudriko P., Nakayiki T. (2022). Risk factors for Crimean-Congo Haemorrhagic Fever (CCHF) virus exposure in farming communities in Uganda. J. Infect..

[41] Atim S.A., Niebel M., Ashraf S., Vudriko P., Odongo S., Balinandi S. (2023). Prevalence of Crimean-Congo haemorrhagic fever in livestock following a confirmed human case in Lyantonde district, Uganda. Parasit. Vectors.

[42] Lule S.A., Gibb R., Kizito D., Nakanjako G., Mutyaba J., Balinandi S. (2022). Widespread exposure to Crimean-Congo haemorrhagic fever in Uganda might be driven by transmission from Rhipicephalus ticks: evidence from cross-sectional and modelling studies. J. Infect..

[43] Nyakarahuka L., Whitmer S., Kyondo J., Mulei S., Cossaboom C.M., Telford C.T. (2022). Crimean-Congo Hemorrhagic Fever outbreak in refugee settlement during COVID-19 pandemic, Uganda, April 2021. Emerg. Infect. Dis..

[44] Sahak M.N., Arifi F., Saeedzai S.A. (2019). Descriptive epidemiology of Crimean-Congo Hemorrhagic Fever (CCHF) in Afghanistan: reported cases to National Surveillance System, 2016–2018. Int. J. Infect. Dis..

[45] Qaderi S., Mardani M., Shah A., Shah J., Bazgir N., Sayad J. (2021). Crimean-Congo Hemorrhagic Fever (CCHF) in Afghanistan: a retrospective single center study. Int. J. Infect. Dis..

[46] Niazi A.U., Jawad M.J., Amirnajad A., Durr P.A., Williams D.T. (2019). Crimean-Congo hemorrhagic fever, Herat Province, Afghanistan, 2017. Emerg. Infect. Dis..

[47] Qaderi S., Hatami H., Omid A.M., Sayad J. (2022). Vaginal bleeding as a sign of Crimean–Congo hemorrhagic fever infection: a case report. J. Med. Case Rep..

[48] Ahmadkhani M., Alesheikh A.A., Khakifirouz S., Salehi-Vaziri M. (2018). Space-time epidemiology of Crimean-Congo hemorrhagic fever (CCHF) in Iran. Ticks Tick-Borne Dis..

[49] Aslani D., Salehi-Vaziri M., Baniasadi V., Jalali T., Azad-Manjiri S., Mohammadi T. (2017). Crimean-Congo hemorrhagic fever among children in Iran. Arch. Virol..

[50] Owaysee Osquee H., Pourjafar H., Taghizadeh S., Haghdoost M., Ansari F. (2017). Laboratory features of 160 CCHF confirmed cases in Zabol of Iran: a 10-year study. J. Infect..

[51] Nili S., Khanjani N., Jahani Y., Bakhtiari B. (2020). The effect of climate variables on the incidence of Crimean Congo Hemorrhagic Fever (CCHF) in Zahedan, Iran. BMC Public Health..

[52] Shahbazi N., Firouz S.K., Karimi M., Mostafavi E. (2019). Seroepidemiological survey of Crimean-Congo haemorrhagic fever among high-risk groups in the west of Iran. J. Vector Borne Dis..

[53] Shahhosseini N., Chinikar S., Shams E., Nowotny N., Fooks A.R. (2017). Crimean-Congo hemorrhagic fever cases in the North of Iran have three distinct origins. Virusdisease.

[54] Baniasadi V., Pouriayevali M.H., Jalali T., Fazlalipour M., Azadmanesh K., Salehi-Vaziri M. (2019). Evaluation of first rapid diagnostic kit for anti-Crimean-Congo Hemorrhagic Fever virus IgM antibody using clinical samples from Iran. J. Virol. Methods.

[55] Hussein S., Qurbani K., Ahmed S.K. (2023). Potential increase in Crimean-Congo hemorrhagic fever incidence in Iraq Post Eid-al-Adha, 2023. New Microbes New Infect..

[56] Al-Abri S.S., Hewson R., Al-Kindi H., Al-Abaidani I., Al-Jardani A., Al-Maani A. (2019). Clinical and molecular epidemiology of Crimean-Congo hemorrhagic fever in Oman. PLoS Negl. Trop. Dis..

[57] Zohaib A., Saqib M., Athar M.A., Hussain M.H., Sial AuR, Tayyab M.H. (2020). Crimean-Congo hemorrhagic fever virus in humans and livestock, Pakistan, 2015–2017. Emerg. Infect. Dis..

[58] Yaqub T., Oneeb M., Mukhtar N., Tahir Z., Shahid F., Subhan S. (2019). Crimean–Congo haemorrhagic fever: case study analysis of a sporadic outbreak from Chakwal, Pakistan. Zoonoses Public Health..

[59] Shahid M.F., Shabbir M.Z., Ashraf K., Ali M., Yaqub S., Ul-Rahman A. (2020). Sero-epidemiological survey of Crimean-Congo Hemorrhagic Fever among the human population of the Punjab Province in Pakistan. Virol. Sin..

[60] Shahid M.F., Shabbir M.Z., Ashraf K., Ali M., Yaqub S., Mukhtar N. (2020). Seroprevalence of Crimean-Congo haemorrhagic fever among three selected risk human groups in disease-endemic region of Pakistan. Zoonoses Public Health.

[61] Rehman K., Bettani M.A.K., Veletzky L., Afridi S., Ramharter M. (2018). Outbreak of Crimean-Congo haemorrhagic fever with atypical clinical presentation in the Karak District of Khyber Pakhtunkhwa, Pakistan. Infect. Dis. Poverty.

[62] Umair M., Khurshid A., Alam M.M., Akhtar R., Salman M., Ikram A. (2020). Genetic diversity and phylogenetic analysis of Crimean-Congo Hemorrhagic Fever viruses circulating in Pakistan during 2019. PLoS Negl. Trop. Dis..

[63] Tariq S., Niaz F., Safi Vahidy A., Qidwai M., Ishaq M., Abbasher Hussien Mohamed Ahmed K. (2023). Crimean-Congo Hemorrhagic Fever (CCHF) in Pakistan: the daunting threat of an outbreak as Eid-ul-Azha approaches. Disaster Med. Public Health Prep..

[64] Umair M., Rehman Z., Haider S.A., Ali Q., Hakim R., Bibi S. (2023). Whole-genome sequencing of Crimean−Congo hemorrhagic fever virus circulating in Pakistan during 2022. J. Med. Virol..

[65] Bower H., El Karsany M., Alzain M., Gannon B., Mohamed R., Mahmoud I. (2019). Detection of Crimean-Congo Haemorrhagic fever cases in a severe undifferentiated febrile illness outbreak in the Federal Republic of Sudan: a retrospective epidemiological and diagnostic cohort study. PLoS Negl. Trop. Dis..

[66] Rahden P., Adam A., Mika A., Jassoy C. (2019). Elevated human Crimean-Congo hemorrhagic fever virus seroprevalence in Khashm el Girba, Eastern Sudan. Am. J. Trop. Med. Hyg..

[67] Ahmed A., Ali Y., Salim B., Dietrich I., Zinsstag J. (2022). Epidemics of Crimean-Congo hemorrhagic fever (CCHF) in Sudan between 2010 and 2020. Microorganisms.

[68] Gambhir R.M., Rathod M.M. (2019). Prognostic factors in CCHF - an Indian origin study. J. Assoc. Physicians India.

[69] Shrivastava N., Kumar J.S., Yadav P., Sharma S., Shete A.M., Jain R. (2022). Development and evaluation of indirect antibody ELISA assay for early diagnosis and surveillance of Crimean-Congo hemorrhagic fever infection in humans. Virus Res..

[70] Mourya D.T., Viswanathan R., Jadhav S.K., Yadav P.D., Basu A., Chadha M.S. (2017). Retrospective analysis of clinical information in Crimean-Congo haemorrhagic fever patients: 2014-2015, India. Indian J. Med. Res..

[71] Mourya D.T., Yadav P.D., Gurav Y.K., Pardeshi P.G., Shete A.M., Jain R. (2019). Crimean Congo hemorrhagic fever serosurvey in humans for identifying high-risk populations and high-risk areas in the endemic state of Gujarat, India. BMC Infect. Dis..

[72] Patel A.A., Dalal Y.D., Parikh A., Gandhi R., Shah A. (2023). Crimean-Congo hemorrhagic fever: an emerging viral infection in India, revisited and lessons learned. Cureus.

[73] Sahay R.R., Shete A.M., Yadav P.D., Patil S., Majumdar T., Jain R. (2021). Sequential determination of viral load, humoral responses and phylogenetic analysis in fatal and non-fatal cases of Crimean-Congo hemorrhagic fever patients from Gujarat, India, 2019. PLoS Negl. Trop. Dis..

[74] Evans T.S., Myat T.W., Hom N.S., Ricks K.M., Maw M.T., Oo Z.M. (2021). Seroepidemiologic survey of Crimean-Congo hemorrhagic fever virus in logging communities, Myanmar. Emerg. Infect. Dis..

[75] Christova I., Panayotova E., Trifonova I., Taseva E., Hristova T., Ivanova V. (2017). Country-wide seroprevalence studies on Crimean-Congo hemorrhagic fever and hantavirus infections in general population of Bulgaria. J. Med. Virol..

[76] Papa A., Markatou F., Maltezou H.C., Papadopoulou E., Terzi E., Ventouri S. (2018). Crimean-Congo haemorrhagic fever in a Greek worker returning from Bulgaria, June 2018. Euro Surveill..

[77] Akhvlediani T., Chitadze N., Chlikadze R., Rostiashvili N., Betashvili M., Imnadze P. (2019). Multivariate relationships between epidemiologic risk factors and zoonotic infections among military personnel in the country of Georgia: a non-linear canonical correlation analysis. Zoonoses Public Health.

[78] Magyar N., Kis Z., Barabás É., Nagy A., Henczkó J., Damjanova I. (2021). New geographical area on the map of Crimean-Congo hemorrhagic fever virus: first serological evidence in the Hungarian population. Ticks Tick Borne Dis..

[79] Abdiyeva K., Turebekov N., Dmitrovsky A., Tukhanova N., Shin A., Yeraliyeva L. (2019). Seroepidemiological and molecular investigations of infections with Crimean–Congo haemorrhagic fever virus in Kazakhstan. Int. J. Infect. Dis..

[80] Head J.R., Bumburidi Y., Mirzabekova G., Rakhimov K., Dzhumankulov M., Salyer S.J. (2020). Risk factors for and seroprevalence of tickborne zoonotic diseases among livestock owners, Kazakhstan. Emerg. Infect. Dis..

[81] Emmerich P., Mika A., von Possel R., Rackow A., Liu Y., Schmitz H. (2018). Sensitive and specific detection of Crimean-Congo Hemorrhagic Fever Virus (CCHFV)—specific IgM and IgG antibodies in human sera using recombinant CCHFV nucleoprotein as antigen in μ-capture and IgG immune complex (IC) ELISA tests. PLoS Negl. Trop. Dis..

[82] Jakimovski D., Grozdanovski K., Rangelov G., Pavleva V., Banović P., Cabezas-Cruz A. (2023). Cases of Crimean-Congo haemorrhagic fever in North Macedonia, July to August 2023. Euro Surveill..

[83] Volynkina A., Lisitskaya Y., Kolosov A., Shaposhnikova L., Pisarenko S., Dedkov V. (2022). Molecular epidemiology of Crimean-Congo hemorrhagic fever virus in Russia. PLoS One.

[84] Dedkov V.G., Shchelkanov M.Y., Bushkieva B.T., Rudenko T.A., Kurdyukova O.V., Galkina I.V. (2017). A neonatal death associated with Crimean-Congo hemorrhagic fever (Republic of Kalmykia, Russia, June 2016). Antiviral Res..

[85] Nikiforova M.A., Kuznetsova N.A., Shchetinin A.M., Butenko A.M., Kozlova A.A., Larichev V.P. (2021). Arboviruses in the astrakhan region of Russia for 2018 season: the development of multiplex PCR assays and analysis of mosquitoes, ticks, and human blood sera. I Infect Genet Evol..

[86] Negredo A., Sánchez-Ledesma M., Llorente F., Pérez-Olmeda M., Belhassen-García M., González-Calle D. (2021). Retrospective identification of early autochthonous case of crimean-Congo hemorrhagic fever, Spain, 2013. Emerg. Infect. Dis..

[87] Negredo A., de la Calle-Prieto F., Palencia-Herrejón E., Mora-Rillo M., Astray-Mochales J., Sánchez-Seco M.P. (2017). Autochthonous Crimean–Congo hemorrhagic fever in Spain. N. Engl. J. Med..

[88] Monsalve Arteaga L., Bellido J.L.M., Lista M.C.V., Santiago M.B.V., Soto P.F., Bas I. (2020). Crimean-Congo haemorrhagic fever (CCHF) virus-specific antibody detection in blood donors, Castile-León, Spain, summer 2017 and 2018. E Euro Surveill..

[89] Monsalve Arteaga L., Muñoz Bellido J.L., Negredo A.I., García Criado J., Vieira Lista M.C., Sánchez Serrano J.Á. (2021). New circulation of genotype V of Crimean-Congo haemorrhagic fever virus in humans from Spain. PLoS Negl. Trop. Dis..

[90] Negredo A., Sánchez-Arroyo R., Díez-Fuertes F., De Ory F., Budiño M.A., Vázquez A. (2021). Fatal case of Crimean-Congo hemorrhagic fever caused by reassortant virus, Spain, 2018. Emerg. Infect. Dis..

[91] Febrer-Sendra B., Fernández-Soto P., García-Bernalt Diego J., Crego-Vicente B., Negredo A., Muñor-Bellido J.L. (2023). A novel RT-LAMP for the detection of different genotypes of Crimean–Congo haemorrhagic fever virus in patients from Spain. Int. J. Mol. Sci..

[92] Pshenichnaya N.Y., Leblebicioglu H., Bozkurt I., Sannikova I.V., Abuova G.N., Zhuravlev A.S. (2017). Crimean-Congo hemorrhagic fever in pregnancy: a systematic review and case series from Russia, Kazakhstan and Turkey. Int. J. Infect. Dis..

[93] Çiçek-Şentürk G., Tekin A., Gürbüz Y., Tütüncü E.E., Sevinç G., Kuzi S. (2019). Retrospective investigation of 9 years of data on needlestick and sharps injuries: effect of a hospital infection control committee. Am. J. Infect. Control.

[94] Beştepe Dursun Z., Korkmaz S., Türe Z., Kaynar L., Dursun A., Çelik İ. (2021). Efficacy of therapeutic plasma exchange in patients with Crimean-Congo hemorrhagic fever. J. Clin. Apher..

[95] Berber I., Korkmaz S., Sarici A., Erkurt M.A., Kuku I., Kaya E. (2021). Therapeutic plasma exchange for envenomation: is it reasonable?. Transfus. Apher. Sci..

[96] Hasanoglu I., Guner R., Carhan A.K., Tufan Z.Y., Caglayik D., Yilmaz G.R. (2018). Dynamics of viral load in Crimean Congo hemorrhagic fever. J. Med. Virol..

[97] Yeşilbağ Z., Karadeniz A., Koçulu S., Kayhan C.B. (2020). Epidemiological characteristics, clinical and laboratory findings supporting preliminary diagnosis of Crimean-Congo hemorrhagic fever in an endemic region in Turkey. Wien. Klin. Wochenschr..

[98] Karakecili F., Cikman A., Aydin M., Binay U.D., Kesik O.A., Ozcicek F. (2018). Evaluation of epidemiological, clinical, and laboratory characteristics and mortality rate of patients with Crimean-Congo hemorrhagic fever in the northeast region of Turkey. J. Vector Borne Dis..

[99] Gürbüz E., Ekici A., Ünlü A.H., Yilmaz H. (2021). Evaluation of seroprevalence and clinical and laboratory findings of patients admitted to health institutions in Gümüşhane with suspicion of Crimean-Congo hemorrhagic fever. Turk J Med Sci..

[100] Yılmaz S., İba Yilmaz S., Alay H., Koşan Z., Eren Z. (2023). Temporal tendency, seasonality and relationship with climatic factors of Crimean-Congo Hemorrhagic Fever cases (East of Turkey: 2012–2021). Heliyon.

[101] Aytekin F.Y., Barut H.Ş., Rüstemoğlu A., Atay A., Günal Ö., Duygu F. (2019). Factors related to fatalities and clinical progression of Crimean-Congo hemorrhagic fever patients and the effects of IL 28-B gene polymorphism. Arch. Virol..

[102] Gülce-İz S., Elaldı N., Can H., Şahar E.A., Karakavuk M., Gül A. (2021). Development of a novel recombinant ELISA for the detection of Crimean-Congo hemorrhagic fever virus IgG antibodies. Sci. Rep..

[103] Karakus N., Yigit S., Duygu F., Barut S., Rustemoglu A., Basol N. (2019). Effects of Paraoxonase-1 variants on course of severity and mortality of Crimean-Congo hemorrhagic fever. Gene.

[104] Gozdas H.T. (2019). Evaluation of Crimean-Congo hemorrhagic fever suspected cases admitted to a secondary care hospital in Kastamonu, Turkey between 2014-2017. Afr. Health Sci..

[105] Fletcher T.E., Leblebicioglu H., Bozkurt I., Sunbul M., Bilek H., Asik Z. (2019). Rotational thromboelastometry alongside conventional coagulation testing in patients with Crimean–Congo haemorrhagic fever: an observational cohort study. Lancet Infect. Dis..

[106] Öztoprak B., Öztoprak İ., Engin A. (2018). Is the brain spared in Crimean-Congo haemorrhagic fever? An MR-SWI study to reveal CNS involvement. Eur. Radiol..

[107] Temocin F., Köse H., Sarı T., Duygu F., Şahin R.O. (2018). Seroprevalence of Crimean–Congo hemorrhagic fever among health care workers in a hospital in an endemic region of Turkey. J. Infect. Dev. Ctries..

[108] Ak E., Gönen M. (2020). A prospective prediction tool for understanding Crimean–Congo haemorrhagic fever dynamics in Turkey. Clin. Microbiol. Infect..

[109] Cosgun Y., Aydemir A., Hedef H., Öz Kamiloglu A., Klemens O., Lattwein E. (2023). Evaluation of nucleoprotein-based enzyme-linked immunosorbent assay for serodiagnosis of acute Crimean-Congo hemorrhagic fever virus infections in a Turkish population. Vector Borne Zoonotic Dis..

[110] Konuk Ş.G., Kılıç R., Yılmaz E.T., Kaya F. (2023). Evaluation of choroidal thickness and choroidal vascularity index in patients with Crimean-Congo hemorrhagic fever. Int. Ophthalmol..

[111] Portillo A., Palomar A.M., Santibáñez P., Oteo J.A. (2021). Epidemiological aspects of Crimean-Congo hemorrhagic fever in Western Europe: what about the future?. Microorganisms.

[112] European Centre for Disease Prevention and Control (2024). https://www.ecdc.europa.eu/en/crimean-congo-haemorrhagic-fever/facts/factsheet.

[113] Nejati J.U., Saghafipour A.U. (2021). Is the reduced incidence of Crimean-Congo hemorrhagic fever related to the preventive measures of COVID-19?. Arch. Clin. Infect. Dis..

[114] Rocha D.C., Santos Louro L., Ewald Oliveira H., Cancian de Araujo B., Ryu S., Vicente C.R. (2025). Use of ProMED as a surveillance system for emerging and re-emerging infectious diseases in Brazil from 2015 to 2020. Viruses.

[115] HabibiSaravi R., Khankeh H., Azar A., Ghasemihamedani F. (2019). Research paper: communicable diseases surveillance system in Iran: strengths and weaknesses 30 years following its implementation. Health Emerg. Disasters Q..

[116] European Centre for Disease Prevention and Control (2019).

[117] Monsalve-Arteaga L., Alonso-Sardón M., Muñoz Bellido J.L., Vicente Santiago M.B., Vieira Lista M.C., López Abán J. (2020). Seroprevalence of Crimean-Congo hemorrhagic fever in humans in the World Health Organization European region: a systematic review. PLoS Negl. Trop. Dis..

[118] Emmerich P., von Possel R., Deschermeier C., Ahmeti S., Berisha L., Halili B. (2021). Comparison of diagnostic performances of ten different immunoassays detecting anti-CCHFV IgM and IgG antibodies from acute to subsided phases of Crimean-Congo hemorrhagic fever. PLoS Negl. Trop. Dis..

[119] Fischer W.A., Weber D.J., Wohl D.A. (2015). Personal protective equipment: protecting health care providers in an ebola outbreak. Clin. Ther..

[120] Fanelli A., Buonavoglia D. (2021). Risk of Crimean Congo haemorrhagic fever virus (CCHFV) introduction and spread in CCHF-free countries in southern and Western Europe: a semi-quantitative risk assessment. One Health.

[121] Freitas N., Legros V., Cosset F.L. (2022). Crimean-Congo hemorrhagic fever: a growing threat to Europe. C. R. Biol..

[122] Bernard C., Holzmuller P., Bah M.T., Bastien M., Combes B., Jori F. (2022). Systematic review on Crimean–Congo hemorrhagic fever enzootic cycle and factors favoring virus transmission: special focus on France, an apparently free-disease area in Europe. Front. Vet. Sci..

[123] Zé-Zé L., Nunes C., Sousa M., de Sousa R., Gomes C., Santos A.S. (2025). Fatal case of Crimean-Congo hemorrhagic fever, Portugal, 2024. Emerg. Infect. Dis..

[124] Anses - Agence nationale de sécurité sanitaire de l'’alimentation, de l'’environnement et du travail Possible Emergence of Crimean-Congo Haemorrhagic Fever in France [internet]. https://www.anses.fr/en/content/possible-emergence-crimean-congo-haemorrhagic-fever-france.

