# Supplementary material for: Human epidemiological data of Crimean-Congo haemorrhagic fever, 2014–2023: scoping review over one decade
Source: One Health. 2026 Oct 13;23:101617. doi: 10.1016/j.onehlt.2026.101617 (PMC13691271; doi:10.1016/j.onehlt.2026.101617)
Supplement: Supplementary file 1 [file mmc1.pdf]

Supplementary File S1

Search string for the PubMed database

Search string for the PubMed database: ("Hemorrhagic Fever, Crimean"[Mesh] OR "Crimean Hemorrhagic Fever"[Tiab] OR "Crimean Congo Hemorrhagic Fever"[Tiab] OR "Crimean Congo Hemorrhagic Fever Virus"[Tiab] OR "Congo Crimean Hemorrhagic Fever"[Tiab] OR "Hemorrhagic Fever Virus, Crimean-Congo"[Mesh] OR "Congo Virus"[Tiab] OR "Congo Fever"[Tiab] OR "CCHF"[Tiab] OR "CCHFV"[Tiab]) AND ("Epidemiology"[Mesh] OR "Epidemiology"[Tiab] OR "Incidence"[Mesh] OR "Incidence"[Tiab] OR "Seroepidemiologic studies"[Mesh] OR "Seroprevalence"[Tiab] OR "Mortality"[Mesh] OR "human infected cases")
