# Supplementary material for: Human epidemiological data of Crimean-Congo haemorrhagic fever, 2014–2023: scoping review over one decade
Source: One Health. 2026 Oct 13;23:101617. doi: 10.1016/j.onehlt.2026.101617 (PMC13691271; doi:10.1016/j.onehlt.2026.101617)
Supplement: Supplementary file 2 [file mmc2.pdf]

Supplementary File S2

Figures

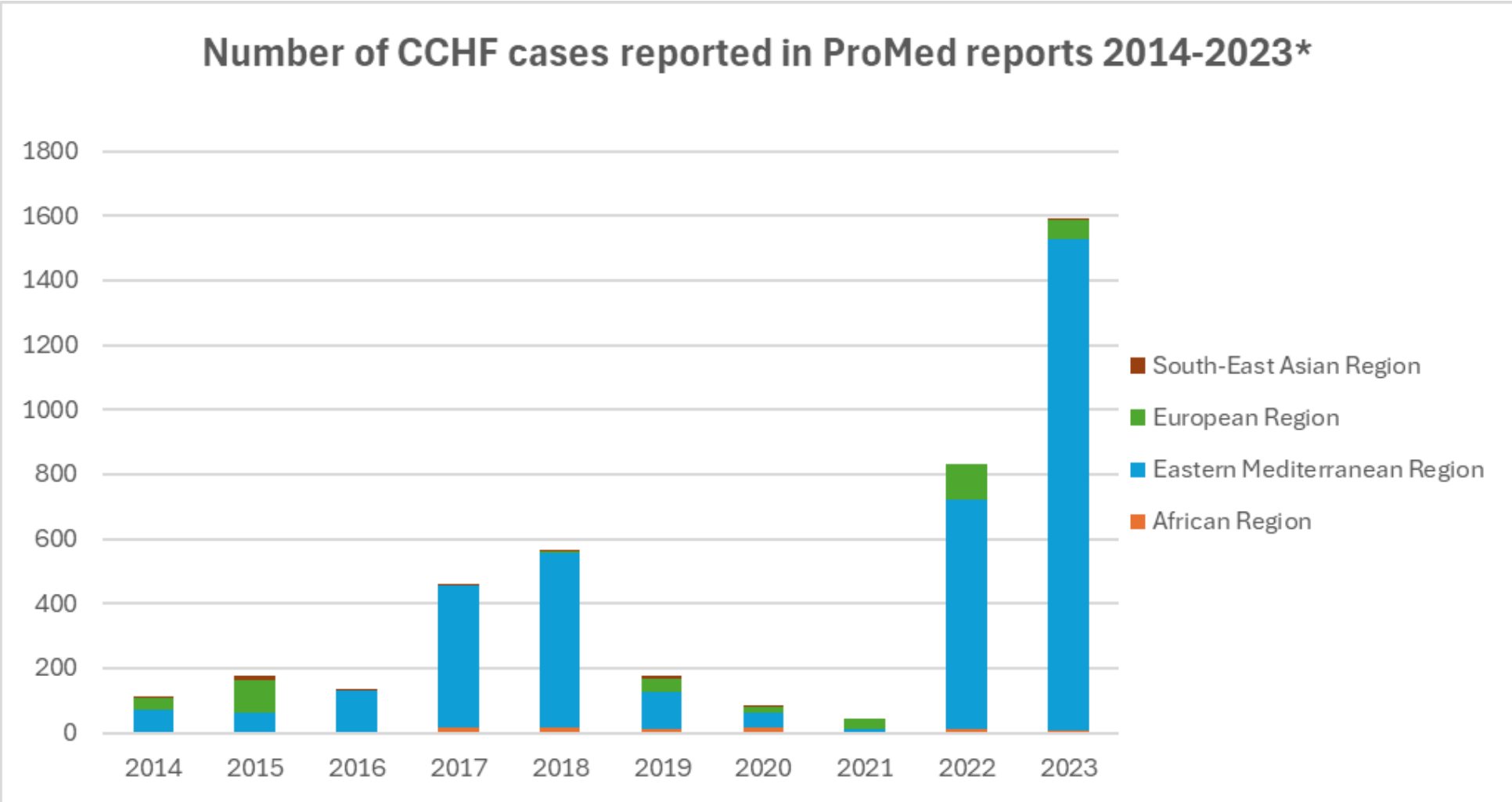

Figure S2.1. Number of CCHF cases reported in ProMED per region per year between 2014-October 9th, 2023

**Publications from the year 2023 were only included until the 9th of October.*

Tables

Table S2.1. Reported mean age of CCHF cases per region extracted from the scientific articles (N=57)

WHO region	*African region*	*Eastern-Mediterranean Region*	*Southeast Asian Region*	*European Region*	*Total*
Number of articles reporting 'mean age' (N)	12	11	3	31	57
Total reported CCHF cases (N)	118	666	40	2,064	2,888
Calculated mean age (years)*	30.1	28.5	36.9	48.0	42.6
Lowest reported mean age (years)	1.5	14.5	22.3	13.0	1.5
Highest reported mean age (years)	62.0	37.7	43.4	74.0	74.0
Median reported mean age (years)	30.0	31.9	N/A	47.6	40.0
25th percentile reported mean age (years)	27.3	26.0	N/A	40.0	30.5
75th percentile reported mean age (years)	45.8	35.4	N/A	54.0	51.1

**Calculated mean age: for each article that had mentioned the 'mean age' of the reported cases, the total number of cases was multiplied by the mean age of that article, which was used to calculate a 'calculated mean age' for the total number of reported CCHF cases in the scientific articles that reported the mean age.*
N/A: not applicable, as too little articles from the Southeast Asian Region reported the age to determine the median, and the 25th and 7th percentile.

Table S2.2. Number of reported CCHF cases, fatalities and mean CFR and CFR range reported in scientific articles (N=49) grouped per study type*

Study groups	*Articles reporting fatalities (N)*	*Cases (N)*	*Fatalities (N)*	*Mean CFR (%)*	*Range CFR (%)*
Case reports or series	19	285	31	10.9	0.0-100
Diagnostic studies	1	190	16	8.4	N/A
Epidemiological studies	13	1,610	297	18.4	0.0-50.0
Observational studies	7	384	46	12.0	3.1-50.0
Outbreak investigations	5	86	24	27.9	0.0-100
Seroprevalence studies	4	194	19	9.8	0.0-11.8
Total	49	2,749	433	15.8	0.0-100

**Of the articles reporting the CFR, their study types are grouped as follows: case reports or series: case report, case report review, case series, case study analysis; diagnostic studies: ecology analysis; epidemiological studies: epidemiological report, epidemiological analysis, molecular epidemiology, prospective epidemiology, retrospective epidemiology; observational studies: prospective cohort, retrospective cohort; outbreak investigations: outbreak investigation; seroprevalence; seroprevalence*

N/A: not applicable, as this study group only includes one article

Table S2.3a. Distribution of fatal and non-fatal CCHF cases per region as reported by ProMED between 2014-October 9th, 2023

Region	*Non-fatal cases (N, (%))*	*Fatal cases (N, (%))*	*Total (N)*	*Subscript group**
African Region	54 (64.3)	30 (35.7)	84	b
Eastern-Meditarranean Region	3212 (88.2)	431 (11.8)	3643	a
European Region	359 (88.9)	45 (11.1)	404	a
Southeast Asian Region	20 (52.6)	18 (47.4)	38	b

**Each subscript letter denotes a subset of regions whose column proportions do not differ significantly from each other at the .05 level (Bonferroni-adjusted).*

Table S2.3b. Chi-square test and pairwise z-test with regional comparison of fatal and non-fatal CCHF cases as reported by ProMED between 2014-October 9th, 2023

Test	*Value*	*df*	*Asymptomatic significance (2-sided)*
Pearson chi-square	85.383	3	<0.001
Likelihood ratio	59.524	3	<0.001
Linear-by-linear association	0.023	1	0.880

Pairwise z-test significance defined at α = .05 (Bonferroni-adjusted)
